# Improving the Lung Cancer Clinical Trial Development by Incorporating Competing Risk Factors

**DOI:** 10.1155/2021/2477285

**Published:** 2021-09-14

**Authors:** Zhu Wenbo, Zhao Qing, Wang Li, Zhu Hangju, Zhang Junying, Han Jing, Qing Rong, Feng Jifeng, Shi Meiqi

**Affiliations:** Jiangsu Cancer Hospital, Affiliated Cancer Hospital of Nanjing Medical University, China

## Abstract

**Introduction:**

Distinct from other diseases, as cancer progresses, both the symptoms and treatments evolve, resulting in a complex, time-dependent relationship. Many competing risk factors influence the outcome of cancer. An improved method was used to evaluate the data from 6 non-small-cell lung cancer (NSCLC) clinical trials combined in our center since 2016 to deal with the bias caused by competing risk factors. *Material and Methods*. Data of 118 lung cancer patients were collected from 2016 to 2020. Fine and Gray's model for competing risk was used to evaluate survival of different treatment group compares with the classic survival analysis model.

**Results:**

Immunotherapy had better progression-free survival than chemotherapy. (HR: 0.62, 95% CI: 0.41-0.95, *p* = 0.0260). However, there were no significant differences in patients who withdrew due to treatment-related adverse events from different groups. (*Z* = 0.0508, *p* = 0.8217). The PD-1/PD-L1 inhibitors in our study did not significantly improve overall survival compared with chemotherapy (HR:0.77, 95% CI:0.48-1.24, *p* = 0.2812), estimated 1-year overall survival rates were 55% and 46%, and 3-year overall survival rates were 17% and 10%, respectively.

**Conclusion:**

When the outcome caused by competing risk exists, the corresponding competing risk model method should be adopted to eliminate the bias caused by the classic survival analysis.

## 1. Introduction

Because of China's large population size, there were 4.51 million new cases of cancers, and the number of deaths was about 3.04 million annually in China [1, 2]. Lung cancer as one of the most common cancers in China has become the most important cause of death, and the age-standardized mortality rate for lung cancer over all cancer registries was 28.49 per 100 000 [3]. At present, the treatment methods of lung cancer in China are surgery, chemotherapy, radiotherapy, and molecular targeted therapy mainly before 2019 [4]. Chemotherapy can cause acute or prolonged immunosuppression in body, which is affected by the type, dosage, and total duration of treatment [5]. Although immunosuppression can prolong a patient's life, it also increases the risk of infection, cardiovascular disease, and others [6]. Especially in oncology clinical trial, cancer patients receiving immunosuppressive therapy such as chemotherapy and radiotherapy were more likely to develop treatment-related adverse events could impact on the dose adjustment and treatment outcome and even can lead to death in severe cases [7]. From 2018, the application of immunotherapy in cancer research is attracting increasing attention from investigators [8]. However, adverse events caused by cancer immunotherapy may be a competing risk for survival endpoints, which have not been thoroughly studied.

In medical research, competing risk mainly refers to the phenomenon that the incidence of target outcome events changes due to the occurrence of other outcome events during the study follow-up period [9]. Distinct from other diseases, as cancer progresses, both the symptoms and treatments evolve, resulting in a complex, time-dependent relationship. Many competing risk factors influence the outcome of cancer clinical trials. For example, treatment-related adverse events or deaths from other noncancer causes may affect the efficacy evaluation. The conclusions drawn without considering competing risks can be biased [10, 11]; however, this issue has obviously not been taken seriously by the lung cancer clinical medicine. In classical statistical methods, the Kaplan-Meier estimate of survival curves and Cox proportional hazard model is widely used to describe survival trends and identify significant prognostic factors. All of these statistical methods consider only one endpoint, which usually is disease progression or death. 9% of patients' treatment-related adverse events (as the censored data in models) led to discontinuation in clinical trial KEYNOTE-042 [12]. In fact, patients who withdrew from the clinical trial due to adverse events may still be able to benefit from the study drug. In other words, compared with the endpoint of interest, the occurrence of the above situation will become a competing risk that reduces the accuracy of model results [13, 14]. Therefore, to assess survival probability where events of interest compete, it is more appropriate to calculate a cumulative incidence function [14].

There has been extensive work focus on addressing the problems posed by competing risk; however, few studies have taken competing risk factors into account in clinical trial design and statistical analysis in the NSCLC field. Therefore, we propose this study to reanalyze completed clinical trials to comprehensively investigate the impact of competing risk factors on the evaluation of drug efficacy and to propose appropriate strategies to deal with competing risk.

## 2. Material and Methods

### 2.1. Patients

From 2016 to 2020, we collected data of 118 lung cancer patients from 6 clinical trials retrospectively (Clinical trial information: NCT02813785; NCT02613507; NCT03085069; NCT02220894; NCT02864394; NCT03134872). All eligible patients were 18 years of age or older and diagnosed with NSCLC, who have received no PD-1 or PD-L1 therapy for their advanced or metastatic NSCLC. They were complied with inclusion/exclusion criteria rigorously. Their compliance was preferable to that of the average patient. A total of 95 patients in both groups died or had disease progression in this study.

### 2.2. Assessments

All imaging obtained on the study had be submitted for a central independent radiologists' reviewer who would assess the images using Response Evaluation Criteria in Solid Tumors (RECIST) 1.1 for determination. The all amendments and protocol were authorized by the ethics committee at our center. The trial was conducted in accordance with the protocol, its amendments, and the standards of good clinical practice. All patients provided written informed consent prior to enrollment.

### 2.3. Outcomes

The primary endpoint was disease progression or death with competing risk factors. Competing risks were also in presence as above, such as the discontinued due to treatment-related adverse events versus disease progression and cancer-specific death versus noncancer-specific death. The relationship between the competing risk and event of interest was evaluated by competing risks regression analysis.

In clinical practice, the symptoms of cancer patients are the focus of the investigator. Especially in lung cancer study, respiratory symptoms were rigorously recorded during each treatment cycle or follow-up. Meanwhile, according to the result of the competing risk model, an extra analysis of respiratory symptoms was evaluated in our study. Besides, the efficacy of different PD-1/PD-L1 inhibitors (pembrolizumab, camrelizumab, nivolumab, and atezolizumab) was evaluated by subgroups.

### 2.4. Statistics and Analysis

Demographic data, such as gender, age, ECOG performance status, and tumor histological features were collected at baseline. Safety was assessed in the treatment population and was defined as all allocated patients receiving at least one dose of study treatment. Adverse events were monitored continuously till 30 days after treatment ended and graded by the CTCAE 4.0.

The Kaplan-Meier estimate of survival curves as the classic method was used to estimate progression-free survival and overall survival data for patients who were lost to follow-up was censored [15]. The Cox proportional hazard model was designed to estimate hazard ratios and subgroups analysis [16].

**Figure 1:**
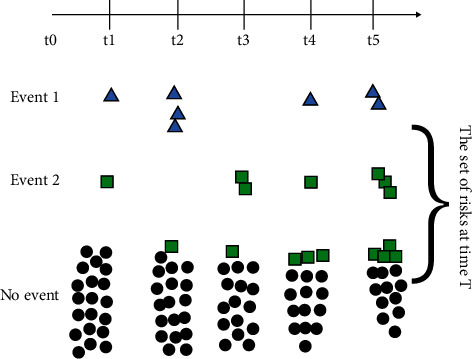
Schematic diagram of Fine and Gray's model.

The competing risk model is an analysis method for dealing with multiple potential outcome data (including competing risk events). These data include the endpoint event that caused the failure ending and the time of failure. There may be multiple endpoint events. These potential end-point events are called competing risk events. In study of competing risk events, if the existence of competing risks are ignored, the correlation between research factors and diseases will be exaggerated or overshadowed. The theoretical basis of the competing risk model is shown in Figure 1 below, where event 1 represents the target outcome event (blue triangle), and event 2 represents the competing event (green square). Different from the traditional calculation of survival rates, the individuals with event 2 occurring before time *T* are retained in the set of risks at time *T* (the green square is still counted in the denominator rather than censoring).

In our study, the discontinued due to treatment-related adverse events was defined as the competing risk rather than censored. Cumulative incidence function for each endpoints mentioned above was compared using Gary's test and multivariate competing risk regression analysis based on Fine and Gray's model [17].

All statistical analyses were performed using SAS 9.3 and *R* software.

## 3. Results

From 2016 to 2018, these 6 NSCLC clinical trials for non-small-cell lung cancer have been carried out in our center. 73 patients were assigned to receive immunotherapy, and 45 patients were assigned to receive chemotherapy [18]. The baseline characteristics were similar between groups (Table 1).

Any grade of treatment-related adverse events occurred in 61 (83.6%) of 73 patients in the immunotherapy group and 41 (91.1%) of 45 patients in the chemotherapy group (appendix1). Grade 3-5 treatment-related adverse events occurred in 19 (26.0%) of 73 patients in the immunotherapy group, and 23 (51.1%) of 45 patients in the chemotherapy grou, was significantly different between two groups (*p* = 0.0057) (Table 2 and Appendix 1). In the immunotherapy and chemotherapy groups, treatment-related adverse events led to drug discontinuation in 7 of 73 patients (9.6%) and 3 of 45 patients (6.7%) (Appendix 1). Adverse events of four PD-1/PD-L1 inhibitors were not same between the intragroup and also different from the chemotherapy group (Table 2). Adverse events of respiratory were specifically recorded in our study. Immunosuppression of immunotherapy is similar to chemotherapy, and there were no significant differences in the incidence of pneumonia, cough, dyspnea, and fever (Table 2 and Appendix 1).

**Figure 2:**
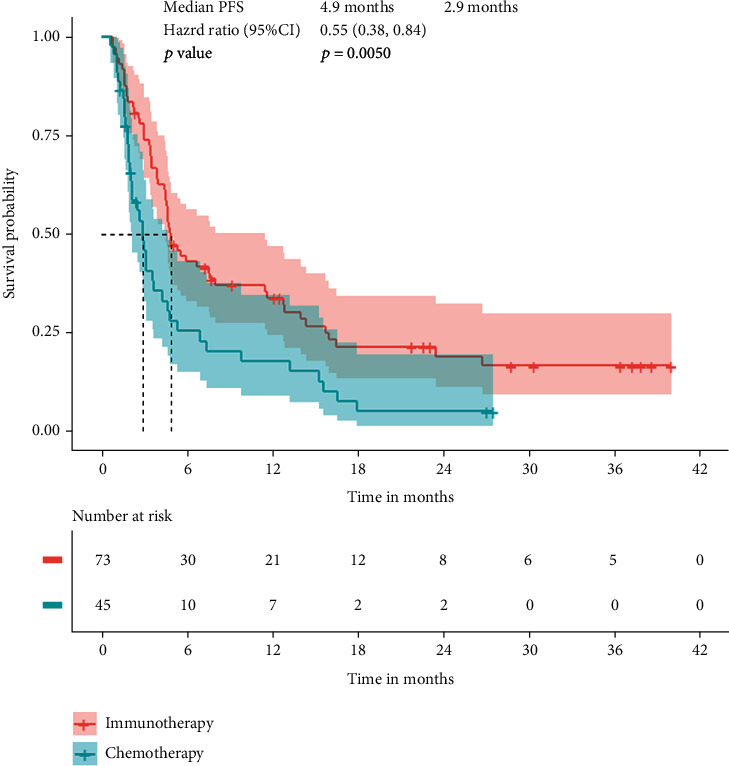
Kaplan-Meier estimate of progression-free survival in all randomized patients.

**Figure 3:**
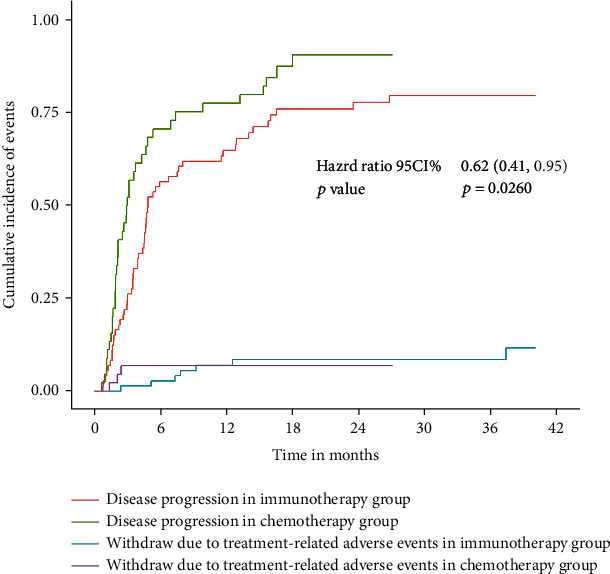
Cumulative incidence of events stratified by disease progression and withdraw due to treatment-related adverse events as competing events for the immunotherapy group and chemotherapy group.

**Figure 4:**
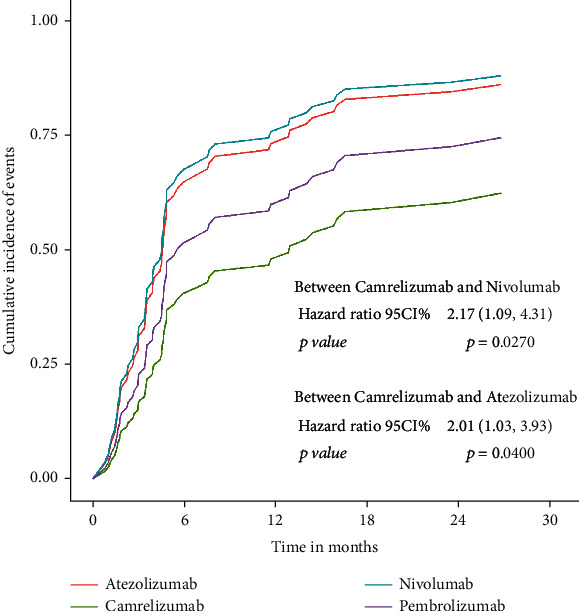
Cumulative incidence of events stratified by atezolizumab, camrelizumab, nivolumab, and pembrolizumab.

A total of 95 patients in both groups died or had disease progression. Median progression-free survival was 4.9 months (95% CI 4.4-7.6) in the immunotherapy group and 2.9 months (95% CI 2.0-4.3) in the chemotherapy group (HR:0.55, 95% CI:0.38-0.84, *p* = 0.0050) (Figure 2). The curve of the cumulative incidence function was showed to compare with the Kaplan-Meier estimate curve in Figure 3 (HR: 0.62, 95% CI: 0.41-0.95, *p* = 0.0260) represented immunotherapy still have better progression-free survival. However, there were no significant differences in patients who withdrew due to treatment-related adverse events from different groups (*Z* = 0.0508, *p* = 0.8217). Subgroup analysis of four was estimated by the competing risk regression model which showed that the camrelizumab had better progression-free survival than nivolumab and atezolizumab in statistic (Figure 4).

**Figure 5:**
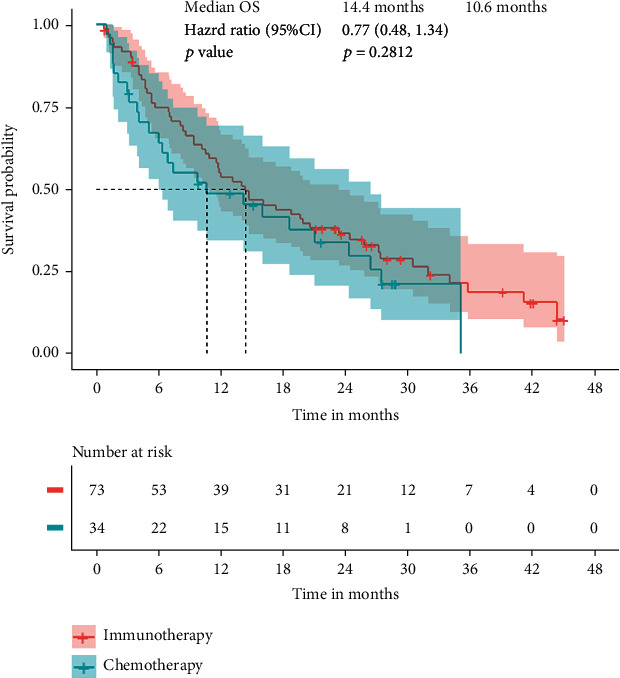
Kaplan-Meier estimate of overall survival in all randomized patients.

During the survival follow-up, we found 11 patients in the chemotherapy group that received immunotherapy after disease progression. So, we have to eliminate these patients from the cohort. The sample size of remaining 107 patients was not enough to get the truly result of overall survival evaluation, median overall survival was 14.4 months (95% CI 10.2-20.6) in the immunotherapy group and 10.6 months (95% CI 5.0-24.3) in the chemotherapy group (HR:0.77, 95% CI:0.48-1.24, *p* = 0.2812) (Figure 5), estimated 1-year overall survival rates were 55% and 46%, and 3-year overall survival rates were 17% and 10%, respectively. Only 1 noncancer-specific death event was observed during the survival follow-up so that we cannot assess the competing risk for overall survival.

## 4. Discussion

The aim of our study was to evaluate the safety and efficacy of 6 NSCLC clinical trials combined in our center since 2016. Traditional disease progression is defined as treatment that was continued until radiographic progression, the patient developed intolerable toxic effects, the investigator decided to stop treatment, or the patient withdrew consent. And on that basis, we added competing risk factor to the cumulative incidence function that helped us to further understand the relationship between competing risk and survival. The result showed that the Kaplan-Meier estimate (HR:0.55, 95% CI:0.38-0.84, *p* = 0.0050) and Fine and Gray's competing risk regression (HR: 0.62, 95% CI: 0.41-0.95, *p* = 0.0260) both represented that immunotherapy significantly prolonged progression-free survival, but the hazard ratio of two is not the same (0.55 vs. 0.62). This distinction is worth considering, and it is possible that the efficacy of PD-1/PD-L1 inhibitors may be overestimated due to the presence of competing risk. Besides, the meaningful results also showed there were no significant differences in patients who withdrew due to treatment-related adverse events from different groups (*Z* = 0.0508, *p* = 0.8217), which again represented when competing risk event occurred, and it would affect the progression-free survival of treatment. So, how to reduce the bias of efficacy evaluation caused by competitive risk will be the problem that this study hopes to solve. In our study, adverse events of respiratory were most factors as the competing risks, such as pneumonia, cough, and dyspnea which were present in the majority of cases. Acute respiratory deterioration after chemotherapy has been reported in previous studies [19]. Besides, cancer treatments can also cause derangements of innate and adaptive immune defense mechanisms responsible for protecting the lungs from infections [20]. Thus, correctly handling or avoiding the deterioration of adverse events of respiratory and infections can help improve the survival and reduce the incidence of competing risk events to make the efficacy evaluation results of clinical trials more accurate finally.

Many studies showed that PD-1 had significant advantages in efficacy over monotherapy, chemotherapy, and targeted therapy without significantly increased adverse event [21]. The safety profile for immunotherapy and chemotherapy in our study showed that treatment-related adverse events (grades 3-5) were not similar from the different PD-1/PD-L1 inhibitors. Consistent with previous studies [12, 22], the incidence of adverse events was lower in the immunotherapy group than in the chemotherapy group. Only one patient died because of the disease progression resulting from adverse events. So, when analyzing the relationship between adverse events and survival, we still keep in mind whether there is a competing risk might overestimate the absolute risk of the event of interest [23]. If the cohort has more than 10% competing risks compare with primary event, using Cox proportional hazard model would no longer be reliable [24]. As an adjusted function, Fine and Gray's competing risk regression was necessary to be used to balance the risk and survival [17].

Compared with chemotherapy, PD-1/PD-L1 inhibitors did not significantly improve overall survival in our study. In our view, OS is difficult to evaluate for long-lived tumors, because patients will go through the treatment, progression, and retreatment. In our study, the number of chemotherapy cohort was eliminated from 45 to 34 because we found 11 patients in the chemotherapy group received immunotherapy after disease progression. As more and more PD-1/PD-L1 inhibitors are marketed, this situation will become increasingly prominent in the future for evaluating the difference between immunotherapy and chemotherapy. Patients in the chemotherapy group in clinical trials naturally seek immunotherapy after disease progression, which will overestimate the overall survival of chemotherapy. This detail should be paid more attention during the follow-up, and the data inclusion and exclusion criteria should have been set in the study design to avoid the above question. The noncancer-specific death was set as the competing risk to cancer-specific death in our study. However, during the survival follow-up, it was difficult for us to know the true causes of death of the patients because the majority eventually died at home. Besides, death caused by cardiovascular disease and other malignancy is easier to identify than death caused by pneumonia and pulmonary disease [25]. Although it failed this time, how to identify the cause of death and reduce the difficulty of obtaining information accuracy will be an important component of our future work.

The PD-L1 expression, as the most widely used predictor, has been studied in many clinical trials. With the deepening understanding of tumor immune escape, it is very necessary to establish a broad framework composed of a variety of biomarkers for patient selection and precision medicine [26]. Furthermore, multitarget combination therapy may be superior to immunotherapy plus chemotherapy, and an antitarget/PD-L1 bispecific antibody might provide a choice for cancer patients resistant to immune checkpoint inhibitors [27]. Clinical trials are currently under way in our center, and it is expected to provide patients with new therapies in the future.

However, there are some limitations to our study. Data from patients in single-center clinical trials are scarce so much that we need to merge data from multiple parallel trials, which consistency will be a challenge to analysis. In the follow-up study, we will further collect the postmarketing data of these PD-1/PD-L1 inhibitors for verification. As mentioned in the previous paragraph, we avoided the data distortion caused by crosstreatment as much as possible, but this also led us to eliminate too much patient from cohort. Thus, this factor should be taken into consideration when calculating the sample size.

## 5. Conclusion

In our study, PD-1/PD-L1 inhibitors did significantly improve progression-free survival and had fewer treatment-related adverse events compared to the chemotherapy group. In competing risk analysis, however, there were no significant survival differences in patients who withdrew due to treatment-related adverse events from different groups, which represented when competing risk event occurred, and it would affect the progression-free survival of treatment. Therefore, when the outcome caused by competing risk exists, the corresponding competing risk model method should be adopted to eliminate the bias caused by the classic survival analysis.

## Figures and Tables

**Table 1:** Baseline characteristic.

	Immunotherapy (*n* = 73)				Chemotherapy (*n* = 45)	*p* value
	Pembrolizumab (*n* = 12)	Camrelizumab (*n* = 23)	Atezolizumab (*n* = 19)	Nivolumab (*n* = 19)		
Characteristic						
Age, median (range)	63 (50-77)	66 (40-75)	67 (43-78)	68 (39-75)	62 (40-75)	0.4158
Men	9	16	16	15	35	0.7747
Women	3	7	3	4	10	
ECOG performance status score						
0	4	6	7	5	15	0.7162
1	8	17	12	14	30	
Tumor histological features						
Squamous	4	8	6	6	14	0.8420
Nonsquamous	8	15	13	13	31	
TNM stage						
III	5	7	7	6	15	0.9189
IV	7	16	12	13	30	

**Table 2:** Treatment-related adverse events (grade 3-5) and adverse events of respiratory (any grades).

	Immunotherapy (*n* = 73)				Chemotherapy (*n* = 45)
	Pembrolizumab (*n* = 12)	Camrelizumab (*n* = 23)	Atezolizumab (*n* = 19)	Nivolumab (*n* = 19)	
Grade 3-5 adverse event occurred					
White blood cell count decreased	0	3 (13%)	0	1 (5%)	13 (29%)
Anemia	0	3 (13%)	1 (5%)	0	1 (2%)
Platelet count decreased	0	3 (13%)	1 (5%)	0	
Neutropenia	0	2 (8%)	1 (5%)	0	8 (18%)
Hyperglycemia	0	1 (4%)	0	0	0
Leucoderma	0	1 (4%)	0	0	0
Hemoptysis	0	1 (4%)	1 (5%)	0	0
Neutrophil count	0	1 (4%)	0	0	14 (31%)
Pneumonia	0	1 (4%)	0	0	0
GGTP rise	1 (8%)	0	0	0	0
Pancreatitis	1 (8%)	0	1 (5%)	0	0
Serum amylase increased	0	0	1 (5%)	0	0
Erythra	0	0	1 (5%)	0	0
Hyponatremia	0	0	1 (5%)	0	0
Bone pain	0	0	1 (5%)	1	0
Myelodysplastic syndrome	0	0	1 (5%)	0	0
Lymphocyte count decreased	0	0	0	1	3 (7%)
Fatigue	0	0	0	1	1 (2%)
Leukopenia	0	0	0	0	5 (11%)
Cough	0	0	0	0	1 (2%)
Abducens nerve disorder	0	0	0	0	2 (4%)
Intestinal obstruction	0	0	0	0	1 (2%)
Zoster	0	0	0	0	1 (2%)
Adverse events of respiratory (any grades)					
Pneumonia	2(17%)	2(9%)	1(5%)	2(11%)	2(4%)
Cough	10(83%)	9(39%)	11(58%)	7(37%)	23(51%)
Dyspnea	0	11(4%)	3(16%)	1(5%)	9(20%)
Fever	0	0	2(11%)	0	0

## Data Availability

The data used to support the findings of this study are available from the corresponding author upon request.

## Supplementary Materials

Supplementary Materials Supplementary Appendix 1 shows the statistical analysis results of treatment-related adverse events.
