# Supplementary material for: Improving the Lung Cancer Clinical Trial Development by Incorporating Competing Risk Factors
Source: Biomed Res Int. 2021 Sep 14;2021:2477285. doi: 10.1155/2021/2477285 (PMC8457938; doi:10.1155/2021/2477285)
Supplement: Supplementary Materials — Supplementary Appendix 1 shows the statistical analysis results of treatment-related adverse events. [file 2477285.f1.docx]

Appendix1

Table 1.1 Treatment-related adverse events of any grade between two groups had no statistical difference.

| Group | Cases occurred | Non-cases | Chi-square | *p* |
| --- | --- | --- | --- | --- |
| immunotherapy | 61 | 12 | χ^2^=1.3537 | *p*=0.2446 |
| chemotherapy | 41 | 4 |  |  |

Table 1.2 Treatment-related adverse events of grade 3-4 between two groups had statistical difference.

| Group | Cases occurred | Non-cases | Chi-square | *P value* |
| --- | --- | --- | --- | --- |
| immunotherapy | 19 | 54 | χ^2^=7.6408 | ***p*=0.0057** |
| chemotherapy | 23 | 22 |  |  |

Table 1.3 Treatment-related adverse events led to discontinuation between two groups had no statistical difference.

| Group | Cases occurred | Non-cases | Chi-square | *P value* |
| --- | --- | --- | --- | --- |
| immunotherapy | 7 | 66 | χ^2^=0.3065 | *p*=0.5798 |
| chemotherapy | 3 | 42 |  |  |

Table 2.1 Cases occurred of Cough between two groups had no statistical difference.

| Group | Cases occurred | Non-cases | Chi-square | *P value* |
| --- | --- | --- | --- | --- |
| immunotherapy | 7 | 66 | χ^2^=1.0458 | *p*=0.3065 |
| chemotherapy | 2 | 43 |  |  |

Table 2.2 Cases occurred of Pneumonia between two groups had no statistical difference.

| Group | Cases occurred | Non-cases | Chi-square | *P value* |
| --- | --- | --- | --- | --- |
| immunotherapy | 27 | 46 | χ^2^=2.2746 | *p*=0.1315 |
| chemotherapy | 23 | 22 |  |  |

Table 2.3 Cases occurred of Dyspnea between two groups had no statistical difference.

| Group | Cases occurred | Non-cases | Chi-square | *P value* |
| --- | --- | --- | --- | --- |
| immunotherapy | 15 | 58 | χ^2^=0.0052 | *p*=0.9427 |
| chemotherapy | 9 | 36 |  |  |

Table 2.4 Cases occurred of Fever between two groups had no statistical difference.

| Group | Cases occurred | Non-cases | Chi-square | *P value* |
| --- | --- | --- | --- | --- |
| immunotherapy | 2 | 71 | * | *p*=0.5241 |
| chemotherapy | 0 | 45 |  |  |

*Exact probability method
